# Phytolith-Occluded Carbon Storages in Forest Litter Layers in Southern China: Implications for Evaluation of Long-Term Forest Carbon Budget

**DOI:** 10.3389/fpls.2019.00581

**Published:** 2019-05-03

**Authors:** Xiaodong Zhang, Zhaoliang Song, Qian Hao, Yidong Wang, Fan Ding, Alin Song

**Affiliations:** ^1^Institute of Surface-Earth System Science, Tianjin University, Tianjin, China; ^2^Tianjin Key Laboratory of Water Resources and Environment, Tianjin Normal University, Tianjin, China; ^3^College of Land and Environment, Shenyang Agricultural University, Shenyang, China; ^4^Institute of Agricultural Resources and Regional Planning, Chinese Academy of Agricultural Sciences, Beijing, China

**Keywords:** forest litter layer, phytolith, phytolith-occluded carbon, carbon sequestration, forest carbon budget

## Abstract

Phytolith-occluded carbon (PhytOC) can be preserved in soils or sediments for thousands of years and might be a promising potential mechanism for long-term terrestrial carbon (C) sequestration. As the principal pathway for the return of organic matters to soils, the forest litter layers make a considerable contribution to terrestrial C sequestration. Although previous studies have estimated the phytolith production fluxes in the above-ground vegetations of various terrestrial ecosystems, the storages of phytoliths and PhytOC in litter layers have not been thoroughly investigated, especially in forest ecosystems. Using analytical data of silica, phytoliths, return fluxes and storages of forest litter, this study estimated the phytolith and PhytOC storages in litter layers in different forest types in southern China. The results indicated that the total phytolith storage in forest litter layers in southern China was 24.34 ± 8.72 Tg. Among the different forest types, the phytolith storage in bamboo forest litter layers (15.40 ± 3.40 Tg) was much higher than that in other forests. At the same time, the total PhytOC storage reached up to 2.68 ± 0.96 Tg CO_2_ in forest litter layers in southern China, of which approximately 60% was contributed by bamboo forest litter layers. Based on the current litter turnover time of different forest types in southern China, a total of 1.01 ± 0.32 Tg of PhytOC per year would be released into soil profiles as a stable C pool during litter decomposition, which would make an important contribution to the global terrestrial long-term biogeochemical C sink. Therefore, the important role of PhytOC storage in forest litter layers should be taken into account in evaluating long-term forest C budgets.

## Introduction

Global warming, as one of the major challenges facing human survival and development, is mainly caused by the rapid increases in greenhouse gas (e.g., CO_2_) concentrations in the atmosphere (IPCC, 2013; Fang et al., 2018). Terrestrial biogeochemical carbon (C) sequestration counteracts about 30% of the total anthropogenic CO_2_ emissions to the atmosphere, and thus plays a crucial role in mitigating long-term climate warming (Law and Harmon, 2011). Currently, one of the most promising mechanisms of terrestrial biogeochemical C sequestration is C occlusion within phytoliths (phytolith-occluded carbon, PhytOC), which has drawn the attention of many researchers (Parr and Sullivan, 2005; Zuo and Lü, 2011; Song et al., 2012b; Li et al., 2013).

Phytoliths, also called plant stones or plant opal, are the silicified features of plants and mainly take the shapes of plant cell walls, cell lumens and the intercellular spaces of the cortex (Piperno, 1988; Parr and Sullivan, 2011). Generally, silicon (Si) in the soil solution is taken up by plant roots in the form of Si(OH)_4_ or Si(OH)_3_O^-^, then transported with the transpiration stream and finally deposited as phytoliths or nanostructures of silica bodies (Ma, 2003; Neumann, 2003). Compared with nanostructures of silica bodies, the size of phytoliths mainly ranges from 5 to 250 μm and phytolith morphotypes generally vary with plant species, which makes them more stable due to their microscale internal cavities (Piperno, 1988; Strömberg, 2004; Lu et al., 2007; Song et al., 2016). Phytolith contents depend not only on plant phylogeny (Hodson et al., 2005), but also on the type of plant tissues and the soil Si availability (Van Soest, 2006; Henriet et al., 2008; Li et al., 2013; Yang X.M. et al., 2015). As phytoliths consist mainly (66–91%) of silica (SiO_2_) and show a positive correlation with Si content in plant (Li B.L. et al., 2014), the phytolith content can be estimated directly or indirectly from plant Si content (Hodson et al., 2008; Parr et al., 2010; Song et al., 2012a, 2013; Anala and Nambisan, 2015).

During the formation of phytoliths, between 0.2 and 5.8% of organic C can be occluded within the phytoliths (Bartoli and Wilding, 1980; Parr et al., 2010; Santos et al., 2010; Li et al., 2013). Relative to other organic C fractions, PhytOC is stable and can persist in the soils at a millennial scale due to the strong resistance of phytoliths to decomposition (Wilding, 1967; Parr and Sullivan, 2005; Zuo et al., 2014). For example, Zhang et al. (2017) have estimated that soil phytolith turnover time in the subtropical and tropical areas ranged from 433 to 1018 years. Previous studies indicated that the PhytOC accumulation rate in tropics and subtropics was 7.2–8.8 kg ha^-1^ yr^-1^, which contributed to nearly 37% of the global mean long-term soil organic carbon accumulation rate (Parr and Sullivan, 2005). In addition, the average turnover time of soil phytoliths ranged from 200 years to longer than 1000 years for most terrestrial ecosystems (Borrelli et al., 2010; Parr et al., 2010; White et al., 2012). Although a fraction of phytoliths may be dissolved, many studies have demonstrated that most phytoliths are stable and could be conserved for hundreds of years. Thus, the potential of phytoliths for the long-term terrestrial biogeochemical sequestration of atmospheric CO_2_ is quite considerable (Parr et al., 2010; Song et al., 2012b).

In global terrestrial ecosystems, approximately 50–90% of the total annual C flux occurs between forests and the atmosphere (Bonan, 2008; Beer et al., 2010), indicating a significant contribution of forests to the terrestrial biogeochemical C cycle (Fang et al., 2002). In China, the area of forest is approximately 2.08 × 10^8^ ha according to the Eighth National Inventory of Forest Resources (Shen et al., 2017; Fang et al., 2018). In terms of geographical distribution, more than 35% of the forest resources in China are distributed in southern region. Previous studies have focused mainly on the production fluxes of phytoliths or PhytOC in above-ground vegetation of various forest types. For example, Song et al. (2013) indicated that the phytolith C sequestration in the above-ground vegetation in China’s forest was about 1.7 Tg CO_2_ yr^-1^, approximately 30% of which was attributed to bamboo due to its high PhytOC production. Li B.L. et al. (2014) indicated that the phytolith C sequestration by bamboo in China was equivalent to 0.29 Tg CO_2_ yr^-1^, approximately 75, 3, and 22% of which was contributed by scattered, mixed and clustered bamboo communities, respectively. However, the contributions of phytoliths and PhytOC in forest litter layers as C storages have not been studied in depth.

As a principal pathway for the return of organic matter to soils, litter layers represent significant C stocks and have a distinct influence on the C dynamics in forest ecosystems (Harmon et al., 1986). Therefore, estimating the phytolith and PhytOC storages in forest litter layers at a regional scale is very essential and would play a significant role in predicting the future evolution of forest C storages under different climatic conditions. In the forests of southern China, previous studies have investigated litter and its C storages in the forest litter layer (Cornwell et al., 2010; Shen et al., 2017; Zhu et al., 2017). However, the extent of the PhytOC storages in forest litter layers and its distribution among different forest types remain unknown, although a few local-scale studies have estimated PhytOC storage in the litter layers (He et al., 2016; Xiang et al., 2016; Ying et al., 2016). In this study, we used data on silica, phytoliths, and forest litter return flux and storage in forests of southern China to estimate the phytolith and PhytOC storages in litter layers in different forest types in southern China, aiming to provide a reference for a future re-evaluation of forest C budgets.

## Materials and Methods

### General Characteristics of the Forest Types in Southern China

In southern China, forests are categorized into six types, according to the principles of Chinese vegetation regionalization (Fang et al., 2002). They are subtropical and tropical coniferous forest (STC), subtropical coniferous and broadleaf mixed forest (SCB), subtropical evergreen and deciduous broadleaf forest (SEDB); subtropical evergreen broadleaf forest (SEB), subtropical and tropical bamboo forest (STB), and tropical monsoon forest (TM) (Table 1). Across the six types of forest, the mean annual temperature (MAT) varies from 2 to 25°C, and the mean annual precipitation (MAP) ranges from 500 to 2000 mm. The main species composition of each forest type in this study are shown in Table 1.

**Table 1:** Properties of dominant forest types in southern China.

Forest type	Area (10^6^ ha)	Altitude (m)	MAT (°C)	MAP (mm)	Main tree species composition
STC	29.54	300–800	8–20	800–1600	*Pinus massoniana, Pinus yunnanensis, Pinus armandii, Cunninghamia lanceolata, Cryptomeria fortunei, Cupressus funebris, Keteleeria fortunei, Amygdalus davidiana, Cathaya argyrophylla, Pinus latteri, Pinus fenzeri-ana*
SCB	4.68	2500–3000	2–14	500–1600	*Tsuga longibracteata*, *Juniperus chinensis, Larix griffithiana, Tilia amurensis, Abies faxoniana*
SEDB	12.48	1000–2200	16–20	800–1600	*Pteroceltis tartarinowii, Sapium sebiferum, Sapium rotundifolia, Liquidambar formosana, Cyclobalanopsis glauca, Cinnamomum calcarea, Fagus longipetiolata, Manglietia chingii*
SEB	21.37	≤2500	15–21	750–2000	*Cyclobalanopsis glauca, Cyclobalanopsis glaucoides, Cyclobalanopsis gracilis, Castanopsis eyrei, Liquidambar formosana, Castanopsis ceratacantha, Liquidambar formosana, Platycarya strobilacea*
STB	7.2	400–800	15–20	1200–1800	*Phyllostachys pubescens, Phyllostachys sulphurea, Phyllostachys bambusoides, Phyllostachys propinqua, Shibataea kumasasa, Sasa argenteastriatus, Dendrocalamopsis oldhami*
TM	0.95	500–700	20–25	1600–2000	*Ostodes paniculatus, Cinnamomum calcarea, Tarrietia parvifolia, Albizia chinensis, Cryptocarya chinensis, Liquidambar formosana, Musa basjoo, Hevea brasiliensis*


*STC, subtropical and tropical coniferous forest; SCB, subtropical coniferous and broad-leaf mixed forest; SEDB, subtropical evergreen and deciduous broad-leaf forest; SEB, subtropical evergreen broad-leaf forest; STB, subtropical and tropical bamboo forest; TM, tropical monsoon forest.*

### Phytolith Content–Silica Content Transfer Function and Phytolith Content Estimation

**FIGURE 1:**
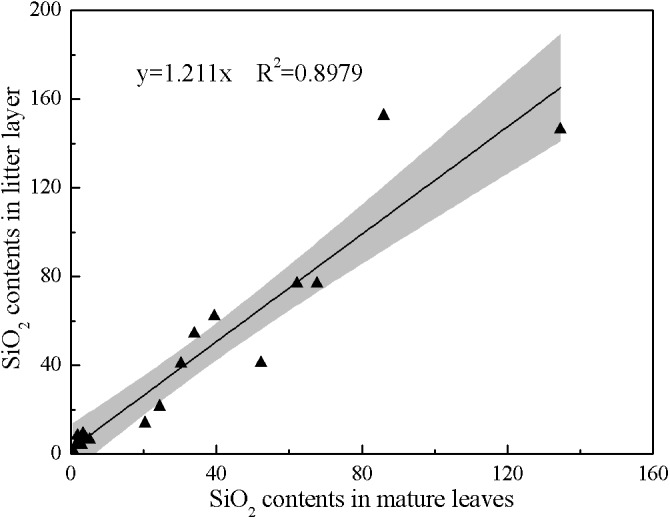
The correlation of SiO_2_ contents in mature leaves and litter layer of different forest types.

The data for SiO_2_ content of mature leaves across different tree species in southern China were collected from published monographs (Hou, 1982; Chen et al., 1997; Song et al., 2013) and theses (He et al., 2016; Ying et al., 2016). To calculate SiO_2_ content in forest litter, we constructed a transfer function (Eq. 1) between SiO_2_ content in mature leaves and in forest litter by a regression analysis method (Figure 1), based on the combined data for SiO_2_ content in the two pools from previous studies (Yang J. et al., 2015; He et al., 2016; Xiang et al., 2016; Ying et al., 2016).

SiO_2_ content in forest litter (wt. %) = 1.211 × SiO_2_ content in mature leaves (wt. %)

$$\left(R^{2}=0.8979,p<0.01\right)$$

As phytoliths consist mainly of SiO_2_ and the phytolith content in litter leaf is generally significantly positively correlated with SiO_2_ content of the leaf litter (Parr and Sullivan, 2005; Song et al., 2012a, 2013), phytolith content of different forest litter layers can be estimated based on the phytolith content-SiO_2_ content transfer function of the samples published in the paper by Song et al. (2013), as follows:

Phytolith content (wt. %) = 0.953 × silica content (wt. %)

$$\left(R^{2}=0.96,p<0.01\right)$$

### Estimation of Phytolith and Return Fluxes and PhytOC Storages in Litter Layers From Different Forest Types

When plants or plant parts die and decay, phytoliths formed in plant tissues can return to the forest floor, along with litter, maintaining their morphological integrity and their chemical characteristics (Strömberg, 2004; McInerney et al., 2011). Therefore, phytolith return flux of litter layers in different forest types can be estimated based on the data of phytolith content and the return flux of forest litter:

phytolith return flux=litter return flux×phytolith content

where phytolith return flux is the weight of phytoliths returned to the floor in a given forest type per area per year (kg ha^-1^ yr^-1^), phytolith content is the content of phytolith in the unit mass of forest litter (wt. %), and litter return flux is the net return flux of forest litter in per area per year (kg ha^-1^ yr^-1^).

Phytolith-occluded carbon content is the organic C content occluded within phytoliths. When the organic materials wrapped on the surface of phytoliths are completely removed and the phytoliths remain intact, the values of PhytOC content are precise. Previous studies indicated that PhytOC content ranges from less than 0.1% to up to 10%, but mainly from 0.2 to 5.8% (Jones and Handreck, 1965; Parr and Sullivan, 2005; Santos et al., 2012; Song et al., 2016). Therefore, in this study, we used a median PhytOC concentration in phytoliths (3%) to estimate the PhytOC storages in different forest litter layers. The PhytOC storages in different forest litter layers were calculated based on the values for litter storage per unit area, phytolith content, PhytOC content and forest area as follows:

PhytOC storage=litter storage per area×phytolith content×PhytOC content×forest area×[44/12]

where PhytOC storage is the total PhytOC amount in each forest litter layer (Tg CO_2_), litter storage per area is the storage of litter in per area of different forest floors (t ha^-1^), phytolith content is the content of phytolith in different forest litter layers and can be estimated by Eq. 2 (wt. %), and forest area is the area of each forest type in southern China (10^6^ ha). The equation is multiplied by [44/12] to transform the data from ‘Tg C’ to ‘Tg CO_2._’

## Results

### Phytolith Concentration in Different Forest Litter Layers

There was a distinct difference in litter SiO_2_ concentration among various plant species (Table 2). Phytolith concentration in forest litters ranged from 0.64 to 203.37 g kg^-1^ across all the different plant species. At the same time, the phytolith concentration of forest litter layers also varied greatly among the different forest types (2.45–148.54 g kg^-1^). Generally, the phytolith concentration of forest litter layers in STB was 148.54 ± 32.77 g kg^-1^, which was the highest among all forest type. In SEDB, SEB, and TM, the phytolith concentration of forest litter layers was 24.54 ± 16.34 g kg^-1^, 17.18 ± 9.49 g kg^-1^ and 20.14 ± 14.56 g kg^-1^, respectively, which were moderate values among the various forest types. The lowest phytolith concentration of forest litter layers were found in STC (2.45 ± 1.21 g kg^-1^) and SCB (4.29 ± 2.30 g kg^-1^).

**Table 2:** SiO_2_ and phytolith contents in litter of dominant tree species from six forest types in southern China.

Forest types	Species	SiO_2_ content in litter (g kg^-1^)	Phytolith content in litter (g kg^-1^)
STC	*Pinus massoniana*	1.96	1.87
	*Pinus yunnanensis*	0.67	0.64
	*Pinus armandii*	3.35	3.19
	*Cunninghamia lanceolata*	2.45	2.34
	*Keteleeria evelyniana*	1.57	1.50
	*Cupressus funebris*	2.44	2.33
	*Cupressus duclouxiana*	3.27	3.12
	*Juniperus formosana*	4.84	4.62
	Average	2.57 ± 1.27	2.45 ± 1.21
SCB	*Larix griffithiana*	8.24	7.85
	*Tilia amurensis*	6.10	5.82
	*Lonicera maximowiczii*	6.10	5.82
	*Picea purpurea*	2.30	2.19
	*Abies faxoniana*	2.97	2.83
	*Abies spectabilis*	1.57	1.50
	*Tsuga chinensis*	4.24	4.04
	Average	4.50 ± 2.41	4.29 ± 2.30
SEDB	*Pteroceltis tartarinowii*	48.87	46.57
	*Celtis sinensis*	49.41	47.09
	*Sapium sebiferum*	62.98	60.02
	*Sapium rotundifolia*	15.99	15.24
	*Liquidambar formosana*	26.04	24.82
	*Quercus fabri*	18.41	17.54
	*Cyclobalanopsis glauca*	16.92	16.13
	*Cinnamomum calcarea*	21.01	20.03
	*Platycarya strobilacea*	8.69	8.28
	*Acer negundo*	17.56	16.74
	*Fagus longipetiolata*	18.17	17.31
	*Acer sinense*	24.34	23.20
	*Manglietia chingii*	6.30	6.00
	Average	25.75 ± 17.14	24.54 ± 16.34
SEB	*Cyclobalanopsis glauca*	25.34	24.15
	*Cyclobalanopsis glaucoides*	23.74	22.62
	*Cyclobalanopsis gracilis*	16.96	16.16
	*Castanopsis eyrei*	9.83	9.37
	*Liquidambar formosana*	37.79	36.01
	*Castanopsis carlesii*	11.84	11.28
	*Castanopsis fargesii*	7.51	7.16
	*Castanopsis fabri*	14.17	13.50
	*Castanopsis hystrix*	9.59	9.14
	*Castanopsis ceratacantha*	12.39	11.81
	*Castanopsis hystrix*	20.35	19.39
	*Quercus fabri*	18.03	17.18
	*Liquidambar formosana*	39.67	37.81
	*Schima wallichii*	8.36	7.96
	*Platycarya strobilacea*	14.90	14.20
	Average	18.03 ± 9.96	17.18 ± 9.49
STB	*Phyllostachys pubescens*	113.36	108.03
	*Phyllostachys vivax McClure*	111.54	106.30
	*Phyllostachys. parvifolia*	119.17	113.57
	*Phyllostachys aureosulcata*	136.25	129.85
	*Phyllostachys bambusoides*	126.80	120.84
	*Phyllostachys bambusoides*	154.42	147.16
	*Phyllostachys heterocycla*	110.70	105.49
	*Phyllostachys propinqua*	153.08	145.89
	*Sinobambusa tootsik*	177.06	168.74
	*Shibataea kumasasa*	143.64	136.89
	*Pseudosasa japonica*	136.98	130.54
	*Pleioblastus kongosanensis*	213.40	203.37
	*Sasa auricoma*	207.95	198.17
	*Sasa argenteastriatus*	149.81	142.77
	*Bambusa multiplex*	175.85	167.59
	*Dendrocalamus latifloru*	206.61	196.90
	*Bambusa tuldoides*	178.27	169.90
	*Dendrocalamopsis oldhami*	190.75	181.78
	Average	155.87 ± 34.39	148.54 ± 32.77
TM	*Ostodes paniculatus*	35.24	33.59
	*Cinnamomum calcarea*	23.37	22.28
	*Tarrietia parvifolia*	4.36	4.16
	*Albizia chinensis*	7.09	6.75
	*Cryptocarya hainanensis Merr*	34.96	33.32
	*Liquidambar formosana*	20.71	19.74
	*Musa basjoo*	41.16	39.23
	*Hevea brasiliensis*	2.21	2.10
	Average	21.14 ± 15.28	20.14 ± 14.56


*SiO_*2*_ contents in forest litter layer are estimated from silica contents of mature leaves using forest silica content transfer function between silica content in litter layer and mature leaves of Eq. 1. At the same time, phytolith contents in forest litter layer are estimated base on forest phytolith content-silica content transfer function of Eq. 2 from Song et al. (2013).*

### The Phytolith Return Fluxes and PhytOC Storages of Forest Litter Layers in Southern China

**FIGURE 2:**
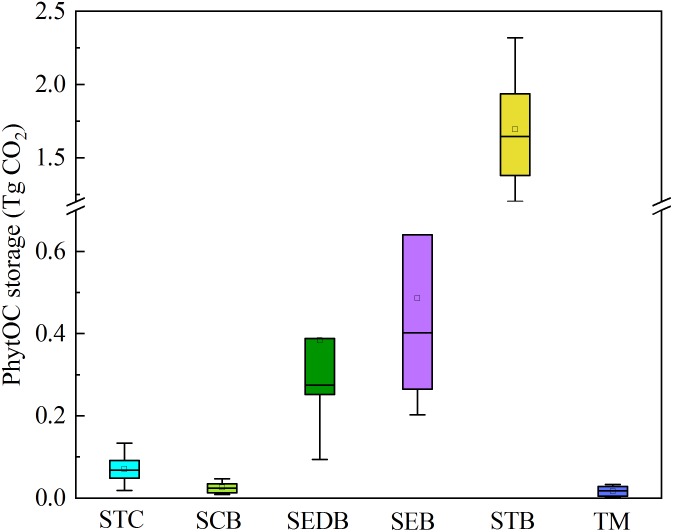
PhytOC storages of forest litter layers in different forest types.

The phytolith return fluxes through forest litter were significantly different among the different types of forest (Table 3). Generally, the mean ± SD phytolith return flux for the six types of forest in southern China was 168.73 ± 58.67 kg ha^-1^ yr^-1^. Phytolith return flux was the highest in STB (484.25 ± 106.93 kg ha^-1^ yr^-1^), moderate in SEDB (154.35 ± 102.94 kg ha^-1^ yr^-1^), SEB (145.34 ± 80.28 kg ha^-1^ yr^-1^) and TM (183.31 ± 132.86 kg ha^-1^ yr^-1^), and lowest in STC (9.94 ± 4.97 kg ha^-1^ yr^-1^) and SCB (35.17 ± 18.81 kg ha^-1^ yr^-1^) (Table 3). Furthermore, the total PhytOC storage in forest litter layer in southern China was 2.68 ± 0.96 Tg CO_2_. Similarly, PhytOC storage was the highest in STBF (1.69 ± 0.37 Tg CO_2_), which contributed to more than 60% of the total PhytOC storage in forests in southern China, followed by SEDB (0.38 ± 0.26 Tg CO_2_) and SEB (0.49 ± 0.27 Tg CO_2_), with the lowest being STC (0.07 ± 0.03 Tg CO_2_), SCB (0.03 ± 0.01 Tg CO_2_), and TM (0.02 ± 0.01 Tg CO_2_) (Figure 2).

**Table 3:** Phytolith return fluxes of forest litter layer in different forest types.

Forest type	Litter return flux^†^ (t ha^-1^ yr^-1^)	Litter storage^†^ (t ha^-1^)	Phytolith return flux (kg ha^-1^ yr^-1^)	Litter turnover time (yr)
STC	4.14 ± 0.21	8.92 ± 1.80	9.94 ± 4.97	2.15 ± 0.43
SCB	8.18 ± 0.34	11.65 ± 2.15	35.17 ± 18.81	1.42 ± 0.26
SEDB	6.30 ± 0.88	11.40 ± 0.97	154.35 ± 102.94	1.81 ± 0.15
SEB	8.45 ± 1.18	12.04 ± 3.60	145.34 ± 80.28	1.42 ± 0.43
STB	3.26 ± 0.49	14.40 ± 2.41	484.25 ± 106.93	4.42 ± 0.74
TM	9.10 ± 0.28	8.17 ± 0.05	183.31 ± 132.86	0.90 ± 0.01


*^†^The data of litter return fluxes and storages in different forest types are from Chen et al. (1997); Peng and Liu (2002), Guan et al. (2004); Lu et al. (2012), Guo et al. (2015); Jia et al. (2016), Ying et al. (2016), and Liu et al. (2017, 2018).*

## Discussion

### Impact of Different Factors on Phytolith Content in Forest Litter Layers

Previous studies have demonstrated that phytolith contents ranged from less than 0.5% in most dicotyledons to more than 15% in some Gramineae, such as bamboo (Epstein, 1994; Hodson et al., 2005; Seyfferth et al., 2013). When plants or plant parts die and decay, phytoliths present in terrestrial plants can be returned to the forest floors in the litters. In this study, the phytolith concentration in forest litter layers varied significantly among different forest types (2.45–148.54 g kg^-1^), due mainly to differences in phytolith return flux, litter decomposition rate and phytolith stability (Parr et al., 2010; Song et al., 2012b, 2016).

Phytolith return fluxes showed significant differences among different forest types (*p* < 0.05), which was due to different litter return fluxes or to different phytolith contents in the litter (Song et al., 2013; Yang X.M. et al., 2015; Table 3). For example, phytolith return flux in the bamboo-dominated STB (484.25 ± 106.93 kg ha^-1^ yr^-1^) was significantly higher than that in other forest types (*p* < 0.05). Song et al. (2013) estimated the phytolith contents in China’s forests using the biogenic silica content-phytolith content transfer function and the results indicated that phytolith concentration in different forests in China ranged from 0.5 to 124.5 g kg^-1^ with the average phytolith concentration in STB (105.2 g kg^-1^) being between four and fifty times higher than that in other forest types (Song et al., 2013). This reflects the high Si content of bamboos. A recent study also indicated that the plant species composition of each forest significantly influenced the production and accumulation of phytoliths (Yang et al., 2018). In this study, the plant species compositions of the six types of forest were distinctly different (Table 1), which would be a major cause of their differences in phytolith return flux among different forest litter layers. From our investigation, there were distinct differences between the litter return fluxes in various forest types in southern China (Table 3), a variable which also plays a significant role in phytolith return flux (He et al., 2016; Ying et al., 2016).

Phytoliths accumulated in forest litter layers could be released into soil profiles by litter decomposition, which has an impact on the phytolith content of the forest litter layer. Therefore, litter decomposition rate is another factor influencing phytolith content in forest litter layers. The drivers of litter decomposition rate are multiple, including the effects of environment, composition of the decomposer communities, and the substrate characteristics of the forest litter (Cornelissen, 1996; Aerts, 1997; Cornwell et al., 2010). Previous studies have shown that the decomposition rate of forest litter can vary under different temperature and moisture conditions, as a result of changes in decomposer community composition and biological activities (Waltman and Ciolkosz, 1995; Liski et al., 2010). In this study, climatic conditions in the different forest types show distinct differences (Table 1), probably contributing to different decomposition rates. Furthermore, the main plant species composition of different forest types under various climate conditions in southern China show fundamental differences (Tables 1, 2), which result in different plant species traits. Previous studies indicated that plant species traits were thought to be a major factor that determined the litter decomposition rates (Cornwell et al., 2010; Li Z.M. et al., 2014; Lu et al., 2017). For example, the nutrient chemistry, stoichiometry and physical features of the forest litter had marked effects on the activity and abundance of microbial decomposers (Melillo et al., 1982). At the same time, differences in the plant species composition of different forest types in southern China, with their associated differences in phytolith content (e.g., the phytolith-rich bamboos dominating the STB) could affect the phytolith content in different forest litter layers (Song et al., 2013).

Phytolith stability is another factor influencing phytolith content in the litter layers from different forests. Previous studies had demonstrated that the phytolith geochemical stability is mainly controlled by phytolith properties and climatic and edaphic conditions (e.g., pH, temperature, moisture, etc.) (Iler, 1979; Bartoli and Wilding, 1980; Li Z.M. et al., 2014). For example, Bartoli (1985) demonstrated that phytoliths from beech leaves had a lower degree of crystallization and a lower Al content than those from pine needles, with beech having a much higher equilibrium concentration of silicic acid (300 μmol Si L^-1^) compared to pine (100 μmol Si L^-1^). Furthermore, previous studies indicated that phytolith dissolution rate may increase with soil pH (Fraysse et al., 2006, 2009). Blecker et al. (2006) estimated the turnover times of soil phytoliths in the Central Great Plains across the bioclimosequence and found a distinct correlation of faster turnover with MAP increasing. In addition, Song et al. (2017) estimated the phytolith stability factors in different forest ecosystems based on the phytolith turnover time, and the results showed that phytolith stability factors in tropical forest, temperature forest and boreal forest ranged from 0.6 to 0.9 (Song et al., 2017). In this study, marked differences in plant species compositions and climatic edaphic conditions among the various forest types contributed to the differences in phytolith stability in the various forest litter layers. Although the phytolith contents in litter layers of different forest types are affected by many factors, such as microbial activity, temperature, moisture and phytolith stability so on, phytolith contents in different plant species play the most important role in controlling phytolith contents in litter layers of different forest types in this study.

### PhytOC Storages in Forest Litter Layers in Southern China

**FIGURE 3:**
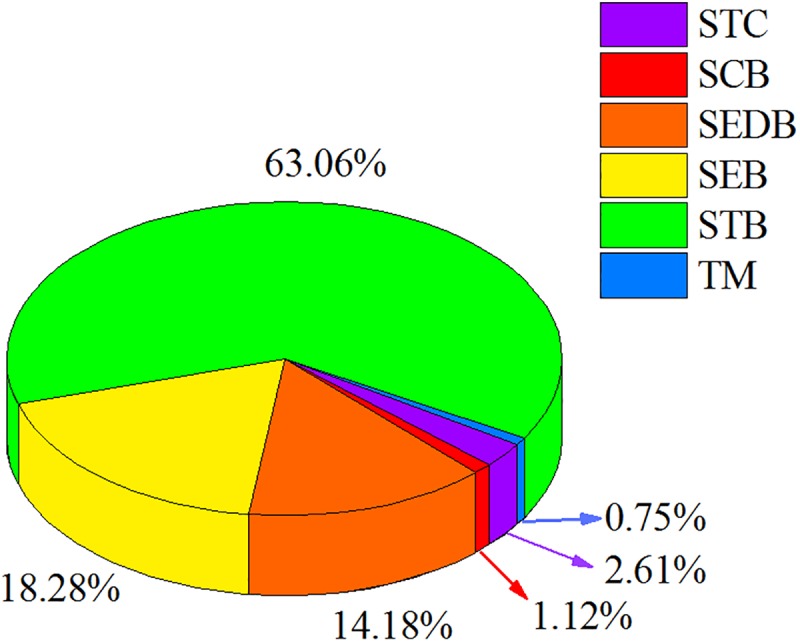
Proportion of PhytOC storage in litter layers of different forests.

**FIGURE 4:**
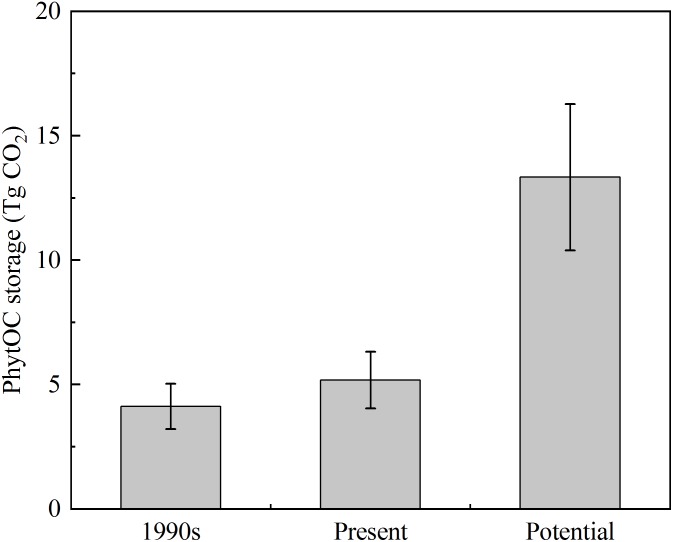
The PhytOC storages of litter layer in the world’s bamboo ecosystem in 1990s, present, and potential.

In this study, there were distinct differences in the litter storages among different forest types due to the differences in stand composition, MAT, MAP, and altitude of each forest type (Table 3). Based on the phytolith contents and forest litter storages in different forest ecosystems, the storages of phytoliths in the various forest types in southern China were calculated and the results showed that the total phytolith storages in forest litter layers in southern China was 24.34 ± 8.71 Tg. Among the different forest types, the phytolith storage of litter layers in bamboo forest (15.40 ± 3.40 Tg) was much higher than that in other forests. Assuming the median concentration of 3% C occluded during the formation of the phytoliths, the total PhytOC storage could reach up to 2.68 ± 0.96 Tg CO_2_ in forest litter layers in southern China, approximately 60% of which was contributed by bamboo forest litter layer but which occupied only 9.5% of the forest area in this region (Figure 2, 3). Global bamboo forest areas in 1990s and now are 1.75 × 10^7^ ha and 2.2 × 10^7^ ha, respectively, and mainly distributed in the subtropical and tropical regions (Liang, 1990; Zhou et al., 2011; Li B.L. et al., 2014). Based on the current PhytOC content in the litter layer of the bamboo-dominated STB and the global bamboo distribution area in 1990s and now, we calculated that the PhytOC storages of litter layers in the global bamboo ecosystem in 1990s and now were 4.12 ± 0.91 Tg CO_2_ and 5.18 ± 1.14 Tg CO_2_, respectively. It is noted that although the forest area in some countries has obviously decreased, the distribution area of bamboo forest in the world has increased at a rate of 3% annually over the last decade and will continue to increase until 2050 due to bamboo afforestation in forest-priority land (Song et al., 2013; Shen et al., 2017). Previous studies have indicated that the global bamboo distribution area will increase from 25 × 10^6^ ha to 100 × 10^6^ ha by 2050, by which point it will occupy approximately 3% of the world’s forest, as a result of bamboo afforestation/reforestation in the subtropical and tropical regions of the world (Zhou et al., 2011; Song et al., 2013). Therefore, based on the rate of increase (3%) of bamboo forest area in the world, the potential size of the global phytolith carbon sink in the litter layer of the bamboo ecosystems would reach up to 13.33 ± 2.94 Tg CO_2_ by 2050 (Figure 4), indicating that bamboo forest will play an increasingly important role in regulating atmospheric CO_2_ sequestration in the form of the bamboo forest phytolith C sink. Although the end result (total PhytOC storage in forest litter layers in southern China) is not fixed and has some uncertainties which are mainly caused by land use changes, litter storages and PhytOC contents in various forest litter, our preliminary estimation is reasonable. In this study, we carefully calculated the minimum, maximum and mean values of PhytOC storages of forest litter layers in different forest types. The results will provide a baseline for evaluating forest carbon budget in the future.

### Implications for Evaluation of Forest Carbon Budget

Phytolith-occluded carbon, as one of the long-term global biogeochemical C sink mechanisms, has attracted the attention of many researchers. Although some phytolith particles (<2 μm) can be quickly dissolved due to their high surface area, approximately 80% of phytoliths released by litter decomposition can be preserved in soils or sediments for 400–3000 years due to their relatively intact surfaces (Parr and Sullivan, 2005; Song et al., 2016; Yang et al., 2018). For example, Zhang et al. (2017) have estimated that soil phytolith turnover time in subtropical and tropical areas ranged from 433 to 1018 years. In the present study, all of the forest types were natural forests with minimal human interference. Assuming that the litter storages in the various forest types have achieved a dynamic balance, the litter turnover time can be estimated from the litter storage size and litter return flux. Results showed that the turnover time in STB (4.42 ± 0.74 years) was the longest, with moderate rates in STC (2.15 ± 0.43 years), SCB (1.42 ± 0.26 years), SEDB (1.81 ± 0.15 years), SEB (1.42 ± 0.43 years), and the shortest rates in TM (0.90 ± 0.01 years), findings which were consistent with the results of Zhang and Wang (2015). Based on the litter turnover time and the size of the PhytOC storage in various forest litter layers, we estimate that a total of 1.01 ± 0.32 Tg CO_2_, in the form of long-term stable organic C components, are released into soil profiles in per year by litter decomposition in southern China. The size of the PhytOC storages in STC, SCB, SEDB, SEB, STB, and TM were estimated to be 0.03 ± 0.02, 0.02 ± 0.01, 0.21 ± 0.01, 0.34 ± 0.19, 0.38 ± 0.08, and 0.02 ± 0.01 Tg CO_2_, respectively. Furthermore, several very large national ecological restoration projects (e.g., Natural Forest Protection Program, the Desertification Combating Program around Beijing and Tianjin, the Sloping Land Conversion Program, etc.) have been implemented to slow climate change and to protect the global environment (Fang et al., 2018), and results show that the national forest litter stock has continuously increased at a steady rate over the past 20 years, mainly due to the expansion of the forest area (Zhu et al., 2017). This implies that increasing amounts of PhytOC will be stored in forest litter layers over the coming decades, while a small proportion of the phytoliths will be dissolved during the decomposition process of forest litter. Therefore, our findings highlight that the PhytOC storage in forest litter layers should be taken into account in the future in any evaluation of the forest C budget, which will play an increasingly important role in the global long-term biogeochemical C sink at a centennial scale.

## Conclusion

In this study we mainly estimated the sizes of PhytOC storages in the litter layers of different forest types in southern China. The results showed that the PhytOC storage was the highest in STB (1.69 ± 0.37 Tg CO_2_), followed by SEDB (0.38 ± 0.26 Tg CO_2_) and SEB (0.49 ± 0.27 Tg CO_2_), with the smallest storages being in STC (0.07 ± 0.03 Tg CO_2_), SCB (0.03 ± 0.01 Tg CO_2_) and TM (0.02 ± 0.01 Tg CO_2_). The total PhytOC storage in forest litter layers in southern China was estimated to be 2.68 ± 0.96 Tg CO_2_, approximately 60% of which was contributed by bamboo forest. In addition, the total amount of PhytOC, as a long-term stable organic C component, released into soil profiles per year by litter decomposition in southern China was estimated to be 1.01 ± 0.32 Tg CO_2_. Based on the current PhytOC content in bamboo litter layers, the potential of the phytolith carbon sink in the world’s bamboo ecosystem litter layers could reach up to 13.33 ± 2.94 Tg CO_2_ in 2050 by practices such as bamboo afforestation/reforestation in the subtropical and tropical regions of the world. Thus, the importance of the PhytOC storage in litter layers of the terrestrial forest ecosystems should be taken into account when evaluating forest C budgets since it plays a significant role in long-term C sequestration operating at centennial-millennial scales.

## Author Contributions

XZ and ZS analyzed the data. QH, YW, FD, and AS contributed to revise it for publication.

## Conflict of Interest Statement

The authors declare that the research was conducted in the absence of any commercial or financial relationships that could be construed as a potential conflict of interest. The handling Editor is currently organizing a Research Topic with one of the authors ZS, and confirms the absence of any other collaboration.
